# Supplementary material for: When calculators lie: A demonstration of uncritical calculator usage among college students and factors that improve performance
Source: PLoS One. 2019 Oct 30;14(10):e0223736. doi: 10.1371/journal.pone.0223736 (PMC6821400; doi:10.1371/journal.pone.0223736)
Supplement: S1 Table — (DOCX) [file pone.0223736.s001.docx]

**Appendix**

| Name | Concrete | Abstract |  |
| --- | --- | --- | --- |
| VP1 | You have just finished eating dinner at a restaurant and the bill is $21. You want to leave a 15% tip. How much would the tip be? | 15% of 21 = _____ |  |
| VP2 | You have four exams in a class. You got an 88, a 90, and an 85 on the first three. What do you have to make on the fourth exam to have at least a 90 in the class overall? | $\frac{88+90+85+x}{4}=90$ |  |
| VP3 | If your grandmother was born in 1942, how old was she in 1994? | What is 1,994 minus 1,942? |  |
| VP4 | You go to Subway and buy a 6" Black Forest Ham Sandwich ($3.75), a 30oz Fountain Drink ($1.80), and a bag of chips ($1.10). After an 8% sales tax, what would the total be? | 3.75 + 1.8 + 1.1 = x  x + (8% of x) = _____ |  |
| VP5 | Shelby is a ULL student who just paid $6,766 in tuition for 4 classes (each 3 credit hours). How much did she pay for each credit hour? | 6,766 divided by 12 = _____ |  |
| VP6 | Kevin's phone bill was $69 this month, but he signed up for a $60 per month plan. He started writing an angry e-mail to a customer service representative and wants to calculate the percent increase as part of his complaint. How much of an increase was there? | Going from 60 to 69 is a _____% increase. |  |
